# Corneal Collagen Cross Linking (CXL) in treatment of Pseudophakic Bullous Keratopathy

**DOI:** 10.12669/pjms.324.10138

**Published:** 2016

**Authors:** Muhammad Saim Khan, Imran Basit, Mazhar Ishaq, Tariq Shakoor, Amer Yaqub, Rana Intisar

**Affiliations:** 1 Dr. Muhammad Saim Khan, MBBS. Armed Forces Institute of Ophthalmology, Rawalpindi, Pakistan; 2 Dr. Imran Basit, FCPS, FRCS (G). Armed Forces Institute of Ophthalmology, Rawalpindi, Pakistan; 3 Prof. Dr. Mazhar Ishaq, FCPS, FRCOphth, FRCSEd, Armed Forces Institute of Ophthalmology, Rawalpindi, Pakistan; 4 Dr. Tariq Shakoor, FCPS. Armed Forces Institute of Ophthalmology, Rawalpindi, Pakistan; 5 Dr. Amer Yaqub, FCPS, FRCSEd. Armed Forces Institute of Ophthalmology, Rawalpindi, Pakistan; 6 Dr. Rana Intisar, FCPS. Armed Forces Institute of Ophthalmology, Rawalpindi, Pakistan

**Keywords:** CXL, Pseudophakic bullous keratopathy, corneal transplantation

## Abstract

**Objective:**

To determine mean change in visual acuity, central corneal thickness and symptoms in patients with pseudophakic bullous keratopathy after treatment with corneal collagen crosslinking.

**Methods:**

This quasi experimental study was conducted at Armed Forces Institute of Ophthalmology, Rawalpindi, Pakistan from April 2015 to Nov 2015. A total of 24 eyes of 24 patients were included in the study. Visual symptoms were graded in five grades (Grade 1-5), Grade-1 being very mild with decreased vision only while patients with all five symptoms (decreased vision, foreign body sensations, pain, watering and photophobia) were graded as Grade-5. Corneal collagen cross linking using topical isotonic riboflavin followed by UVA radiations (3mW/cm^2^ for 10 minutes) was performed in all the patients. Visual acuity (VA), visual symptoms and central corneal thickness (CCT) were recorded before and 04 weeks after the treatment.

**Results:**

A total of 24 eyes of 24 patients (18 male and 6 females) underwent surgery. Age of the patients ranged from 55 to 75 years with mean age 65.83 + 3.89 years. Mean visual acuity was 2.09 + 0.23 before treatment while after treatment it was 2.13 + 0.22. Mean CCT as measured by optical pachymetry (Galilae G6) was 753.96 + 55.16 and 641+ 29.25 before and after surgery respectively. Improvement of clinical symptoms was seen in all the patients.

**Conclusion:**

Corneal collagen cross linking is a temporary but effective symptomatic treatment of pseudophakic bullous keratopathy.

## INTRODUCTION

Pseudophakic bullous keratopathy (PBK) is an important, visually significant, long term complication after cataract surgery that can render the patient legally blind. It results from dysfunction and loss of corneal endothelial cells leading to corneal edema, corneal opacification and epithelial bullae formation. Incidence of bullous keratopathy is 1-2% in various parts of world.^1^-^3^ Patient usually present with decreased vision initially but later on there is intense discomfort, pain, watering, irritation, photophobia due to rupture of bullae and exposure of corneal nerves. Symptomatic patients are commonly managed with topical hypertonic saline, mild topical steroids and bandage contact lens. However, keratoplasty is the eventual surgical management for improving visual outcome in eyes with good visual potential and isolated corneal disorders. Corneal grafting is a costly procedure, not readily available for all the patient and it is not a good treatment option for those cases where there is guarded visual prognosis.^4^-^6^ In such cases various treatment modalities have been tried for symptomatic relief such as PTK (phototherapeutic keratectomy), conjunctival rotation or free flaps, puncturing anterior corneal stroma, and transplantation of amniotic membrane.^7^

Corneal collagen cross linking (CXL) with Riboflavin and ultraviolet A (UVA) radiations is photochemical process that was introduced by Seiler and Spoerl at university of Dresden for treatment of corneal ectatic disorders such as keratoconus and post LASIK ectasias.^8^ The proposed mechanism of action is that riboflavin absorbs UVA light which results in production of free oxygen redicals. These highly reactive oxygen redicals then induce cross linking of corneal stromal collagen and strengthen the cornea.^9^

Over the past few years, CXL has been used in various corneal disorders other than keratoconus such as non-healing corneal ulcer, PBK and Fuch`s endothelial dystrophy.^10^,^11^ However, there is no established evidence of effectiveness of CXL in these conditions. Some authors claim its benefit in non-healing ulcer and symptomatic PBK while others consider this treatment ineffective.^12^,^13^

The rationale of conducting this study is to observe the effect of CXL in patients with symptomatic PBK in our population because corneal grafting is not readily available and if at all possible, most of the patients cannot afford the cost.

## METHODS

This Quasi experimental study was conducted at Armed Forces Institute of Ophthalmology, Rawalpindi, Pakistan from April 2015 to October 2015. A total of 24 eyes of 24 patients suffering from pseudophakic bullous keratopathy were included in the study. Age of the patients ranged from 55-75 years. Those patients who were asymptomatic, had history of trauma, herpetic eye disease, bullous keratopathy secondary to other causes like Fuch`s endothelial dystrophy, advanced glaucoma were excluded from the study. Informed written consent was taken and detailed clinical examination of all the patients fulfilling the inclusion criteria was performed. It included unaided visual acuity and slit lamp examination. Visual symptoms were graded in five grades (Grade 1-5), Grade-1 being very mild with decreased vision only while patients with all five symptoms (decreased vision, foreign body sensations, pain, watering and photophobia) were graded as Grade-5. CXL with isotonic riboflavin was performed on all the patients following standard protocol. Visual acuity (VA), visual symptoms and central corneal thickness (CCT) were recorded before and 04 weeks after the treatment.

### Procedure

All the patients underwent surgery under topical anesthesia. CCT was measured before surgery with optical pachymetery (Galilae G4 and Reichert, Angeled probe pachymeter). Central corneal epithelium was removed with the help of sterile blunt spatula followed by instillation of riboflavin eye drops (1 drop every 2 minutes) for 30 minutes. UVA radiations were delivered using (Vario, CCL-365) for 10 minutes at power settings of 9mW/cm^2^. Bandage contact lens was placed at the end of procedure and patients were advised with topical moxifloxacin, nepafenac and cyclopentolate eye drops three times a day for 01 week. Patients were reviewed at day 1, after 02 weeks and then 04 weeks.

### Statistical analysis

Statistical package for social sciences (SPSS 21.0) was used for statistical analysis. Continuous variables such as age, VA and CCT were described in terms of mean ± SD (standard deviation) and analyzed statistically by paired sample t test while grading of symptoms was analyzed by frequency distribution and Wilcoxon test to determine the significance level (p≤0.05).

## RESULTS

A total of 24 eyes of 24 patients (18 male and 6 females) underwent surgery. Age of the patients ranged from 55 to 70 years with mean age 65.83 ± 3.89 years. Mean visual acuity was 2.09 ± 0.23 before treatment while after treatment it was 2.13 ± 0.22. Mean CCT as measured by optical pachymetry (Galilae G4) was 753.96 ± 55.16 and 641± 29.25 before and after surgery respectively (Table-I). The magnitude of induced change in visual acuity was statistically insignificant, while there was significant change in CCT (Table-II). Grading of clinical symptoms and their frequencies before and after treatment is shown in Table-III. None of the patient had complications such as corneal ulcer and persistent epithelial defect.

**Table-I:** Mean and Standard deviation.

	N	Mean ± SD
Age	24	65.83±3.89
Pre CXL Visual acuity	24	2.09±0.23
Pre CXL Central corneal thickness	24	753.96±55.16
Post CXL Visual acuity	24	2.13±0.22
Post CXL Central corneal thickness	24	641±29.25

**Table-II:** Induced change in CCT and VA.

	Pre treatment	Post treatment	Induced change	P value
Visual Acuity	2.09 ± 0.23	2.13 ± 0.22	0.038 ± 0.16	0.26
Central corneal thickness	753.96 ± 55.16	641± 29.25	112.75 ± 52.32	0.01

**Table-III:** Grades of clinical symptoms before and after CXL.

	Grade 1	Grade 2	Grade 3	Grade 4	Grade 5
Pre CXL	0 (0 %)	0 (0 %)	3 (12.5 %)	8 (33.3%)	13 (64.2%)
Post CXL	16 (66.6%)	8 (33.3%)	0 (0%)	0 (0%)	0 (0%)

**Fig.1:**
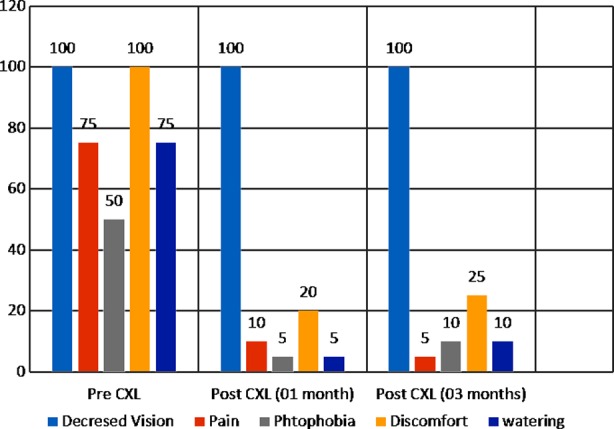
Percentage of symptoms in pre CXL and post CXL groups.

## DISCUSSION

The role of CXL is well established in the treatment of keratoconus, however in recent years this modality has been tried in treatment of PBK. Wollensak and his colleagues observed the possible effectiveness of CXL in PBK and proposed the formation of chemical bonds between collagen fibrils that creates a mechanical barrier and reduce the edema.^14^,^15^ Various other studies also concluded the effectiveness of CXL in symptomatic treatment of PBK, however the effects are transient with recurrence of edema and bullae formation within few months.^9^,^10^,^11^,^16^,^17^

Despite using a different protocol with UVA radiations of 9mW/cm^2^ for 10 minutes instead of 3mW/cm^2^ for 30 minutes (Dresden protocol), we, in our study found out a remarkable and statistically significant improvement in the symptoms as well as CCT of patients at 01 month (Table II & III) which was also concluded by Wollensak et al. and Sharma N et al. in their studies.^9^,^10^,^11^ Some authors such as Ghanem et al. in their series of 14 patients and Arora et al. in 24 patients also found out a significant improvement in the visual acuity in addition to symptomatic relief.^18^,^19^ However, like Sharma et al, we could not find a statistically significant improvement in visual acuity and corneal clarity. The probable reason may be the severe visual loss and relatively advanced stage of disease at the time of presentation in our patients.

The transient effect of CXL in PBK has been claimed by almost all the authors in their studies. The primary reason for this is obviously the endothelial decompensation which is not catered for with CXL. Therefore, the persistent influx of aqueous subsequently overcome the strengthening effect of CXL within couple of months leading to corneal edema, increased thickness, bullae formation and recurrence of symptoms. To overcome this, various techniques are used to increase and prolong the effectiveness of CXL by using concurrent preoperative hypertonic glucose (40%) or glycerol (70%). Authors have concluded positive results with these modified techniques.^10^,^20^

Keratoplasty is the gold standard treatment for PBK, however, donor corneas are not readily available in our part of the world and patients usually have to wait for a period of about 8-12 months. Furthermore, patients who have good vision in the other eye are usually more concerned about the discomfort, pain and watering in the affected eye rather than decreased vision. Therefore, the effect of CXL on PBK which are claimed to be transient are nevertheless significantly effective as a temporary measure especially for patients awaiting definite treatment with corneal transplant.

We think our findings are important and comparable to other published literature, however, the long term effect of CXL on PBK and their subsequent effect on corneal grafting requires study on a larger cohort and a longer follow up.
